# Notes and News

**Published:** 1888-02-18

**Authors:** 


### Notes and News.

Collected and Communicated.

The Southport Convalescent Home has received a legacy of £2,786, under the will of Mr. George Redford, of Southport.

The Workington Infirmary has, during the first year of its existence, received £600 through the weekly contributions of the workmen of the district, who seem desirous of making the institution a sort of provident hospital, and practically proving their right to its benefits.

At a meeting of the committee of Carlisle Infirmary, Miss Allen, of Liverpool, was elected Matron in succession to Mrs. Baber. The new Matron was trained at King's College Hospital, London, and also spent some time at Netley. There were fifty-four candidates for the post. Miss Allen entered upon her duties on the 17th inst.

The managers of the Kent and Canterbury Hospital are now engaged in studying the problem, How to maintain a hospital in full efficiency with a deficit in the income of £500 a-year. It would be interesting if Mr. Layard would supplement his recent article in the *Nineteenth Century* on the art of living on £700 a-year with one that would help the solution of this rather more difficult question.

A general court of governors of the City of London Lying-in Hospital was held on the 8th inst., at the hospital, City Road, under the presidency of Mr. J. B. Morland. The committee of management, in presenting the 137th annual report, expressed their gratification in the satisfactory character of the year's working, both as regards the sanitary condition of the hospital and its financial position. During the year 356 women have been delivered in the hospital, as against 283 in 1886. Three hundred and fifty-eight children were born, viz., 177 boys and 181 girls, seven children were stillborn, and one woman has died, the death of another (from extraneous causes) being the only one since May, 1886, out of 506 deliveries, the mortality being under two per 1,000. The daily average number of beds occupied has been 15·5, and the average stay of each patient has been fifteen days. The out-patients attended in the year numbered 1,204. The employment of midwife pupils has been attended with great success, and the training school for midwives and monthly nurses continues in a prosperous condition, more women being instructed in the hospital during 1887 than in any previous year. The accounts presented showed that, including £241 brought forward, the income from all sources amounted to £3,215, whilst the expenditure had been £2,723, leaving a balance at the bankers of £491. The annual subscriptions showed an increase of £28. The report and accounts were unanimously adopted, and the other formal business having been transacted, cordial votes of thanks were passed to the committee of management, staff, and to Mr. R. A. Owthwaite, the able secretary, general testimony being borne to the admirable conduct of the affairs of the hospital.

Dr. J. Lockhart Livingstone has been appointed House Surgeon to the Bristol Hospital for Sick Children.

The number of in-patients treated at Stroud Hospital during last year was 224, while the out-patients numbered 2,665. The income for the year showed an increase of £73 15*s.* 0½*d.*, and the expenditure a decrease of £27 1*s.* 3*d.*

Dr. Alexander Wallace has been appointed Physician to the Essex and Colchester Hospital at a salary of £200 a-year. Dr. Wallace has long been a resident practitioner in Colchester. There were seven candidates for the appointment.

The members of the Nelson Dramatic Club, which has already distributed £500 among various charities, gave a performance at St. George's Hall, Langham-place, for the benefit of St. John's Hospital for Skin Diseases, on the 31st ult. The performance consisted of the comedy *Garrick*, and a new comedietta entitled *Love and Halfpence.* There was a large and appreciative audience.

Subscriptions are coming in slowly, but in substantial sums, towards the projected Cottage Hospital in Falkirk. The Earl of Zetland has promised a donation of £100, and the men employed in Port Downie Iron Works (Messrs. Walker, Hunter, and Co.'s) have voluntarily agreed to give 6*d.* per quarter each towards the funds of the hospital, the money to be deducted regularly from their wages by the cashier of the firm.

At the annual meeting of the Whitehaven and West Cumberland Infirmary, Mr. Pearson complained that a patient who had received £1,000 as compensation for the injury which had been treated at the infirmary, gave as a donation a sum equivalent to about one-half per cent. of the sum he received. It was not generous; but hospital managers elsewhere have learned to be thankful for mercies quite as small, and not to be surprised when they receive none.

A governor of the Dorset County Hospital writes to the *Dorset County Chronicle* suggesting that in order to reduce the debt on the hospital, each subscriber should add a guinea to the amount of his subscription. "A Governor" proposes doing so himself, and says that the man who can give one guinea can give two. If he cannot easily afford it, let him save the amount in some luxury—a daily glass of wine, or anything—in order to prevent the usefulness of the hospital being curtailed.

Mr. Henry J. Fase, Chaplain to the Westminster Hospital, makes an appeal through the newspapers for clothing for convalescent patients there. He states that boots and shoes are specially useful. Everyone who has gone much among the poor must know how severely the boot question strains the family finances; while those who are connected with School Boards can bear witness how often the defaulting parent's plea for Tommy's non-attendance at school is that his boots were worn out, and he could not go with bare feet in the rain, or the frost, or the snow.

Miss Wood, the Lady Superintendent of the Hospital for Sick Children, Great Ormond-street, declares that it is a popular delusion that every woman is "born a nurse." Many have the will to tend the sick, but few have the skill to do it well. Unhappily, every woman does not possess as a birthright the six qualifications which Miss Wood holds to be fundamentally necessary in a nurse. These are: Presence of mind, gentleness, accuracy, memory, observation, and forethought. They are qualities which have a marked value elsewhere than in the sick room, and if we thought it would bestow them on the sex at large we should incline to wish that every woman was "born a nurse," or, failing that, was trained as one.

THE following vacancies are announced this week, the amount of salary in each case being given after the name of the institution :—

**Resident Medical Officers.**

Birmingham Children's Hospital. Assistant Resident Medical Officer.—£40 per annum, with board.
Birmingham General Hospital. Assistant House Surgeon.
East Suffolk Hospital, Ipswich. Assistant House Surgeon.
Earlswood Asylum for Idiots, Redhill.—£500 per annum.
Liverpool Northern Asylum. Assistant House Surgeon.—£70 per annum, with board.
Menston Asylum, near Leeds. Medical Superintendent.—£400 per annum, with board.
Roxburgh District Asylum, Melrose. Assistant Medical Officer. £80 per annum, with board.
Royal Surrey County Asylum, Guildford. House Surgeon.—£80 per annum, with board.
Staffordshire General Infirmary. Assistant House Surgeon.
York County Hospital. Senior House Surgeon.—£100 per annum, with board.
York Dispensary. Three Resident Medical Officers.—£130 per annum.

**Non-resident Medical Officers.**

Athlone Union. Medical Officer, Private Dispensary.—£140 per annum, and fees.
Bristol Royal Infirmary. Dental Surgeon.
Cancer Hospital, Brompton. Pathologist.—£80 for twelve months.
Forest Hill Provident Dispensary. Medical Officer.
Metropolitan Hospital, Kingsland Road, E. Ophthalmic Surgeon.
National Orthopædic Hospital. Surgical Registrar and Anæsthetist.—£20.
Nenagh Union. Medical Officer, Silvermines Dispensary.—£100 per annum, and fees.
Oldcastle Union. Medical Officer, Oldcastle Dispensary District. —£135 per annum, and fees.
Oughterard Union. Medical Officer, Oughterard Dispensary.—£112 per annum, and fees.
Royal Hospital for Diseases of the Chest, City Road. Assistant Physician.

**Nurses.**

Institution of Trained Nurses, Leicester. Several, Medical, Surgical, and Monthly
Beckett Hospital, Barnsley One for Medical and Surgical Wards.—£25.
Kingston Nurses' Home, Baker Street, Hull. One for Medical, Surgical, and Monthly.
Bradford Eye and Ear Hospital. One, trained.—£25, and uniform.
Nursing Institution, Northampton. One, experienced Surgical.
Workhouse Infirmary Association, 6, Adam Street, Adelphi. Several.

**Probationers.**

Royal Sea Bathing Infirmary, Margate. Several, Lady.
Cromer Cottage Hospital. One, working.

---

THE Hospital Sunday collections at Sheffield amount this year to £2,008 1*s*. 7*d*., but as the collections in some churches have been deferred for a few weeks, a further amount of £70 or £80 may be looked for. It is not expected, however, that the sum of this year's contributions will equal that of 1887, which was £2,341 2*s*. 9½*d*. But considering the long strain on the prosperity and charity of the town which the small-pox epidemic has caused, the sum already received bears a fair testimony to the generous feeling still in Sheffield.

---

Two women, out-patients at the surgical department of the Walsall Cottage Hospital, having complained that they had been improperly treated, Sister Ellen has written to the *Walsall Observer* and to the clerk to the Board of Guardians, explaining the true circumstances of each case. Each of the women came first to the hospital at a time when there was no surgeon in attendance. Sister Ellen attended to the one, Sister Sophia to the other, and each bade the patient return the following day to be seen by the surgeon. This they failed to do, probably from fear that he would cause them some pain, and so injuries which had been bound up in a manner that was meant to be only temporary, and which could easily have been cured, were aggravated by neglect into permanent disablement. No blame can attach to the sisters, each of whom did more for the patients than could strictly be demanded of her ; the fault lies with the patients themselves, who did not obey the directions given, and are now complaining of the consequences of their wilfulness.

A GENERAL meeting of the British Nurses' Association was held on Monday at St. George's Hall, Langham Place. Mr. Savory, President of the Royal College of Surgeons, took the chair. Her Royal Highness the Princess Christian was also present, and there were also on the platform Sir Dyce Duckworth, Sir Henry Acland, Sir Joseph Fayrer, Dr. Matthews Duncan, and others. The attendance of nurses was very large. Her Royal Highness the Princess Christian briefly addressed the meeting. Mr. Savory, in a lucid and eloquent speech, commended to the audience the advantages of union and co-operation for mutual progress. Miss Wood addressed the meeting at some length, and expressed the opinion that as matters stood at present in the nursing world, a nurse might arrive at a house, with a box on the roof of a four-wheeled cab, and say she was a trained nurse, and at the same time know no more about nursing than the horse which drew her there. Miss Mollett also spoke, and explained that the Association was not a benevolent scheme or a charitable association ; but that nurses were asked to combine in order to form themselves into a legally authorized profession. All nurses who joined the Association would be entitled in future to be registered when a charter should have been obtained, but the Association would not take upon itself the right to register nurses. Sir Henry Acland, at the close of the meeting, moved, in graceful terms, a vote of thanks to the Princess Christian for her presence.

---

THE Montrose Royal Asylum bears the pleasant name of Sunnyside, and appears to deserve it. Not only are the certificates of the inspectors such as must give great satisfaction to Dr. Howden and the other officials of the asylum, but a magazine published there and largely written by the patients, shows that they are as happy as their mental condition permits them to be, and are more interested in matters of public interest than might have been expected.

---

"TOMMY ATKINS," writing from Netley Hospital, expresses his regret that Mr. Roberts, a Scripture reader, who has till lately spent his time among the sick soldiers, cheering the sick and consoling the dying, has lately been placed upon "half-pay and half time." The society through whose instrumentality Mr. Roberts was sent to Netley is in want of funds, and can no longer keep him there ; so "Tommy Atkins" appeals to the public of Hampshire and elsewhere to supply the necessary means to enable him and those generally classed under his pseudonym to retain their spiritual comforter and friend.

---

UNDER the heading of "Red Tape at the Lying-In Hospital," a story was told in an evening contemporary a few days ago of a poor woman who was taken in labour in the street, and having been carried to the Lying-In Hospital, in Endell Street, was refused admittance because the officials were not sure whether she was married or not. Of course if such a circumstance had really happened it would have been disgraceful. We are informed, however, on the best authority, that the tale is absolutely destitute of foundation. It appear that the poor woman actually was confined in the street, but that none of the crowd had the presence of mind either to carry her to the hospital, which was close at hand, or to inform any of the officials of the fact. Had those in charge been informed, the case would have been at once received, and the question of its compliance with the regulations would have been inquired into afterwards. Some newspapers seem never to be so happy as when they can discover and proclaim to all the world that their fellow-countrymen have done something or other which proves them to be the basest of human beings. It is a laudable object to make a newspaper interesting ; it is still more laudable to do as one would be done by, and tell the simple truth.

